# 引言

**DOI:** 10.3724/SP.J.1123.2022.08027

**Published:** 2022-10-08

**Authors:** Xiaojia HUANG

灵敏、可靠地检测复杂样品中痕量、超痕量目标物对分析测量学提出了巨大挑战,目前市场上虽已有诸多智能化的现代分析检测仪器,但考虑到复杂的样品基底及目标组分的低含量,在进行仪器分析检测前,需进行有效的样品制备,以降低复杂样品基底的干扰,提高检测准确性,同时对目标组分进行富集,从而改善检测灵敏度。可以说,样品制备过程是整个分析过程中最为重要和耗时的步骤,影响着分析结果的准确性和可靠性。

目前污染物层出不穷,待测样品日益复杂,已有诸多研究发展了形式多样的样品制备技术,但各类技术均有相应的适用范围和目标物,同时存在一定缺陷,因此,发展有效的样品制备新方法、新材料和新技术仍是分析科学领域今后的研究热点。

有鉴于此,受《色谱》期刊委托,我们组织了“样品制备新方法及相关技术”专辑,荣幸邀请到国内部分相关研究人员为本刊撰稿,经过专家严格评审,最终筛选出14篇研究论文、5篇专论与综述和1篇微型述评,涉及磁固相萃取、固相微萃取、分散固相微萃取、QuEChERs和加速溶剂萃取等萃取模式在环境监测、农产品质量安全保障和生物样品分析中的应用。

本专辑文章分别安排在第10期和第11期发表,希望能为相关研究人员提供帮助和带来启迪,提升我国样品制备方法及相关技术的研究水平。

本专辑客座主编

厦门大学环境与生态学院

黄晓佳 教授


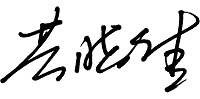


2022年8月30日

